# Danger- and pathogen-associated molecular patterns recognition by pattern-recognition receptors and ion channels of the transient receptor potential family triggers the inflammasome activation in immune cells and sensory neurons

**DOI:** 10.1186/s12974-015-0239-2

**Published:** 2015-02-03

**Authors:** Giorgio Santoni, Claudio Cardinali, Maria Beatrice Morelli, Matteo Santoni, Massimo Nabissi, Consuelo Amantini

**Affiliations:** School of Pharmacy, Section of Experimental Medicine, University of Camerino, Camerino, 62032 Italy; Department of Molecular Medicine, Sapienza University, Rome, 00185 Italy; Department of Medical Oncology, AOU Ospedali Riuniti, Polytechnic University of Marche, Ancona, 60126 Italy; School of Biosciences and Veterinary Medicine, University of Camerino, Camerino, 62032 Italy

**Keywords:** Innate immunity, Inflammasome, IL-1β, Caspase-1, PAMPs, DAMPs, PRRs, TLRs, Ion channels, TRP channels

## Abstract

An increasing number of studies show that the activation of the innate immune system and inflammatory mechanisms play an important role in the pathogenesis of numerous diseases. The innate immune system is present in almost all multicellular organisms and its activation occurs in response to pathogens or tissue injury via pattern-recognition receptors (PRRs) that recognize pathogen-associated molecular patterns (PAMPs) or danger-associated molecular patterns (DAMPs). Intracellular pathways, linking immune and inflammatory response to ion channel expression and function, have been recently identified. Among ion channels, the transient receptor potential (TRP) channels are a major family of non-selective cation-permeable channels that function as polymodal cellular sensors involved in many physiological and pathological processes.

In this review, we summarize current knowledge of interactions between immune cells and PRRs and ion channels of TRP families with PAMPs and DAMPs to provide new insights into the pathogenesis of inflammatory diseases. TRP channels have been found to interfere with innate immunity via both nuclear factor-kB and procaspase-1 activation to generate the mature caspase-1 that cleaves pro-interleukin-1β cytokine into the mature interleukin-1β.

Sensory neurons are also adapted to recognize dangers by virtue of their sensitivity to intense mechanical, thermal and irritant chemical stimuli. As immune cells, they possess many of the same molecular recognition pathways for danger. Thus, they express PRRs including Toll-like receptors 3, 4, 7, and 9, and stimulation by Toll-like receptor ligands leads to induction of inward currents and sensitization in TRPs. In addition, the expression of inflammasomes in neurons and the involvement of TRPs in central nervous system diseases strongly support a role of TRPs in inflammasome-mediated neurodegenerative pathologies. This field is still at its beginning and further studies may be required.

Overall, these studies highlight the therapeutic potential of targeting the inflammasomes in proinflammatory, autoinflammatory and metabolic disorders associated with undesirable activation of the inflammasome by using specific TRP antagonists, anti-human TRP monoclonal antibody or different molecules able to abrogate the TRP channel-mediated inflammatory signals.

## The inflammasome

The innate immune system plays an important role in the host’s first line of defense against microbial infections and involves the recognition of pathogen-associated molecular patterns (PAMPs) or danger-associated molecular patterns (DAMPs) by pattern recognition receptors (PRRs) [1,2]. Activation of PRRs by microorganisms, toxins, chemical compounds, cytoplasmic PAMPs and/or endogenous DAMPS triggers intracellular signaling leading to inflammasome generation.

The PRR family of the innate immune system comprises: the membrane-bound Toll-like receptors (TLRs), C-type lectin receptors, retinoid acid-inducible gene-1-like receptors, and NOD-like receptors (NLRs; nucleotide-binding oligomerization domain receptors) [3].

The TLR family comprises type I transmembrane proteins with an extracellular binding domain composed of leucine-rich repeats and intracellular Toll-interleukin (IL)-1 receptor domains required for signals transduction [4,5]. Based on the TLR cellular localization and PAMP ligands, this family is divided into two groups. The first members (TLR3, TLR7, TLR8, and TLR9) are located in the endoplasmic reticulum (ER), endosomes and lysosomes; the second (TLR1, TLR2, TLR4, TLR5, and TLR6) are expressed on cell membranes and sense different PAMPs, including fungal cell wall components, lipopolysaccharide (LPS), bacterial flagella and peptidoglycan [6,7]. Upon activation, TLRs recruit a set of adaptor molecules such as MyD88 and Toll-IL-1-receptor domain-containing adaptor inducing interferon-β, and promote signaling events leading to the secretion of proinflammatory mediators [8].

The NLR family controls the inflammatory and immune responses via the activation of different signaling pathways involved in nuclear factor (NF)-κB activation (first signal) and caspase-1-mediated cleavage of IL-1β and IL-18 cytokines (second signals) [9]. NLR proteins share highly conserved structures and are divided into several subfamilies based on the nature of their N-terminal domains. NOD1 and NOD2 are two well-characterized members of non-inflammasome NLRs, which mediate NF-κB and mitogen-activated protein kinase activation in response to different PAMPs [10].

Inflammasomes are involved in defensive responses, dangerous inflammation and in pyroptosic cell death [11]. Structurally, the inflammasome is composed of the intracellular adaptor protein ASC (apoptosis-associated speck-like protein containing a CARD) that recruits caspase-1 to the inflammasome complex and of a “sensor NLR” that functions as a molecular scaffold for the activation of caspase-1 for the final secretion of IL-1β and IL-18 [12]. Upon binding to the inflammasome, caspase-1 is cleaved and activated, leading to cleavage of different targets and causing maturation and secretion of the pro-inflammatory IL-1β. Several types of inflammasomes have been described containing different sensor proteins such as NLRP1, NLRP3, NLRP6, NLRP12, NLRP6, retinoid acid-inducible gene-1, AIM-2 and IPAF [9]. Among the NLR inflammasomes, the NLRP3 (nucleotide-binding domain leucine-rich repeat containing family, pyrin domain containing 3) is the most studied, and is activated by a broad range of signals of pathogenic, endogenous and environmental origin. NLRP3 is also activated by endogenous danger signal molecules released as a consequence of tissue injury, such as hyaluronan, extracellular ATP, uric acid crystals and amyloid-β fibrils, industrial particles and nanoparticles such as alum, asbestos, silica and titanium dioxide [9,13]. The mechanisms that have been suggested to mediate NLRP3 triggering are: potassium efflux, reactive oxygen species (ROS) production and alteration in the phagolysosomal [14,15] and lysosomal cathepsins activity [16]. In addition, the NLRP3 inflammasome regulates insulin signaling, the plaque formation in atherosclerosis, myocardial ischemia-reperfusion injury and neurodegeneration [17-19]. Recent data strongly support an important role played by the ion family of Transient Receptor Potential (TRP) channels that mediates interchanged and bidirectional contact and signaling with known PRRs, PAMPs and DAMPs in triggering the first and second signals responsible for inflammasome activation [20].

## The TRP channel family

TRP channels are a family of non-selective cation channels that function as polymodal sensors in different physiological [21] and pathological processes [22]. The TRP channels are membrane proteins with six putative transmembrane segments (S1–S6) and a cation-permeable pore region between S5 and S6. About 30 different mammalian TRP channels are identified and classified into six subfamilies on the basis of sequence homology: TRP canonical (TRPC; TRPC1-7), TRP vanilloid (TRPV; TRPV1-6), TRP melastatin (TRPM; TRPM1-8), TRP polycystin (TRPP; TRPP2, TRPP3, TRPP5), TRP mucolipin (TRPML; TRPML1-3), and TRP ankyrin (TRPA; TRPA1) [23]. The different TRPs show distinct cation selectivity and gating mechanisms [24], and they can be opened by membrane depolarization, ligand binding, or G-protein coupled signaling [25].

TRPCs consist of proteins related to the “canonical” Drosophila TRP; functionally, the TRPC receptor family has been implicated in hypertension, cardiac hypertrophy, vascular inflammation, progressive kidney failure and cancer [26-29].

The TRPV channels are thermo- and chemo-sensitive channels composed of six members [30]. TRPV1 can be activated by a diverse range of stimuli, including membrane depolarization, noxious heat, vanilloid, endocannabinoids, extracellular protons and inflammatory mediators [31]. TRPV1 plays a pivotal role in pain and neurogenic inflammation [20]. Moreover, a role of this receptor in glioblastoma and bladder cancer has been reported [32,33]. TRPMs show high permeability to Ca^2+^ and Mg^2+^ [34,35].

Among TRPMs, TRPM1 functions as a tumor suppressor, and TRPM3 seems to be involved in etiology of amyotrophic lateral sclerosis [34]. TRPM2, TRPM4, and TRPM5 are reported to be sensitive to high temperatures. The best understood cold-sensitive TRP channel is TRPM8, which is activated when the temperature drops below about 20°C [35].

The TRPML subfamily consists of three members characterized by a large E1 loop, between S1 and S2, with several N-glycosylation sites.

The TRPP subfamily has three members, TRPP2, TRPP3, and TRPP5; mutations in TRPP2 lead to autosomal dominant polycystic kidney disease [36].

The TRPA1 channel is the only member of the TRPA subfamily; it is involved in pain and in chronic visceral inflammation [37].

A plethora of diseases are interconnected with alteration of the expression and function of TRP channels. This review focuses attention on the aspects of the interaction between TRP channels and PRRs and PAMPs/DAMPs in mediating inflammasome generation and triggering an inflammatory response.

## The TRP receptors recognize environmental exogenous and endogenous danger signals released during trauma or tissue injury

Noxious stimuli and inflammatory recognition pathways trigger activation of different receptor types shared by cells of the immune and peripheral nervous systems. In particular, TRP channels recognize exogenous signals, DAMPs from the environment (for example, heat, acidity, chemicals) and endogenous danger signals released during trauma/tissue injury (for example, ATP, osmotic stress, uric acid, hydroxynonenals). Other types of cellular stress such as mechanical stress can be also sensed by TRP channels and can induce inflammation [38].

Local extracellular acidosis may promote inflammation at ischemic and inflammatory sites. Acidic extracellular pH triggers NLRP3 inflammasome activation and IL-1β secretion in human macrophages and represents a novel danger signal alerting the innate immunity [39]. The NLRP3 inflammasome complex triggers caspase-1-mediated maturation and secretion of IL-1β, one of the key proinflammatory cytokines. Rajamäki and colleagues have demonstrated that human macrophages exposed to pH 7.5 to 6.0 trigger a pH-dependent secretion of IL-1β and activation of caspase-1 through a mechanism involving the TRPV1 and K^+^ channels. Acidic extracellular pH causes rapid intracellular acidification and IL-1β-inducing effects. Moreover, the NLRP3 knockdown abolishes IL-1β secretion at acidic pH. Remarkably, alkaline extracellular pH inhibited IL-1β response to different NLRP3 activators. Overall these data suggest that acidic environment represents an endogenous danger signal alerting the innate immunity. Low pH contributes to the start of inflammation in acidosis-associated pathologies such as atherosclerosis, post-ischemic inflammatory responses and cancers [39]. Intracellular acidification evoked by extracellular acidosis is regulated through TRPV1 [40]; thus a role for this receptor in the transduction of acidic danger signals activating the inflammasome might be suggested.

TRPM8 receptor is differentially expressed in human immune cells and in primary sensory neurons [41,42]. Increased transcription of IL-1α, IL-1β, IL-6, and IL-8 cytokine genes in human lung epithelial cells through activation of a TRPM8 variant by exposure to cold temperatures has been reported [43]. Moreover, in a recent abstract presented at the ISSAID Congress 2013 by Stoffels and colleagues it has been hypothesized that *in vivo* cold exposure could result in a modulated inflammatory response through activation of temperature-sensitive ion channels [44]. Thus, exposure to cold, or *ex vivo* stimulation of peripheral blood mononuclear cells with menthol, a TRPM8 agonist, induces an exaggerated (local and systemic) cytokine inflammatory response, as evaluated at protein level by enzyme-linked immunosorbent assays in several human-derived cell lines, primary cells and skin biopsies. Activation of the temperature-sensitive TRPM8 channel via TLRs results in an enhanced IL-1β secretion and, likely, NLRP3 inflammasome formation [44].

Exposure to particulate crystals can induce oxidative stress in phagocytes, which triggers NLRP3 inflammasome-mediated IL-1β secretion, activating undesiderable inflammatory responses associated with both autoinflammatory and metabolic diseases. Although mitochondrial ROS have been found to play a central role in NLRP3 inflammasome activation, how the ROS signal causes NLRP3 assembly of the inflammasome is still unknown. Recently, it has been demonstrated that liposome activation of the NLRP3 inflammasome requires mitochondrial ROS. Stimulation of macrophages with liposomes/crystals induces ROS-dependent calcium influx via the TRPM2 channels, and macrophages deficient in TRPM2 display impaired NLRP3 inflammasome activation and IL-1β secretion [45,46]. In this regard, *Trpm2*^−/−^ mice were resistant to crystal-/liposome-induced IL-1β-mediated peritonitis *in vivo*. Moreover, TRPM2-deficient mice are also susceptible to *Listeria monocytogenes* infection [47], a known activator of NLRP3 inflammasome [48]. Therefore, targeting TRPM2 may be effective for treatment of NLRP3 inflammasome-associated inflammatory disorders. Recently, a close interaction between ER and mitochondria in response to NLRP3 inflammasome stimuli has been demonstrated [49]. Interestingly, Murakami and colleagues have showed that ER-derived calcium is essential for mitochondria to maintain a damaged state [50]. Mitochondrial damage induces release of mitochondrial DNA into the cytosol, which activates the inflammasome [51,52]. ROS released from damaged mitochondria activate the TRPM2 channel, which results in calcium influx across the plasma membrane. Thus, it appears that calcium mobilization represents a critical step during NLRP3 inflammasome activation. Calcium is a key signal for several cellular proteases, protein kinases and phospholipases involved in NLRP3 inflammasome activation. These calcium-dependent enzymes may proteolytically regulate the assembly of the NLRP3 inflammasome.

TRPA1 seems to be involved in the detection of reactive chemicals, including environmental irritants such as tear gas and industrial isothiocyanates; it is also activated during tissue injury by endogenous 4-hydroxynonenal and prostaglandins [53,54]. A role for this receptor in the inflammasome activation is in evaluation.

## The role of TRPM7 and TRPV2 channels in osmotic stress and regulatory volume decrease responses in microglia

Changes in cell volume represent a specific signal in the regulation of important physiological processes [55,56]. Cells exhibit rapid volume-regulatory mechanisms to recover their functions in response to different signals. Alterations in osmosensing or osmosignaling are associated with ischemia, hyponatremia, hypothermia and intracellular acidosis [55,57]. Hypotonicity has been found to induce processing and release of IL-1β [58]. Low osmolarities stimulate macrophage swelling and a decrease in intracellular K^+^ and Cl^−^ levels, which activates a regulatory volume decrease (RVD) that is sensed by the NLRP3 inflammasome [59]. The RVD process is controlled by TRPM7 and TRPV2 channels expressed in macrophages. During RVD, TRPV2 is translocated to the plasma membrane where it induces permeabilization [60]. Hypotonicity induces lysosome destabilization and it is sensitive to a broad range of TRP channel blockers that suppress caspase-1 activation and IL-1β release. TRPV2 functions as an osmosensor, and TRPM7 as a controller of the RVD process [61-63]. Blocking TRPM7 function using high extracellular Mg^2+^ concentrations delays RVD, decreases cell permeabilization [64], and reduces IL-1β release in macrophages. In the THP-1 monocytic cell line, TRPV2 gene silencing decreases IL-1β release in response to hypotonicity, without affecting RVD [60]. A decrease in extracellular osmolarity induces phosphorylation and activation of TAK1 (transforming growth factor-β activated kinase-1) in the HeLa cell line [65]. Inhibition of TAK1 phosphorylation abrogates hypotonicity-induced caspase-1 activation and IL-1β processing and activation of TAK1 is abolished in the presence of BAPTA-AM, a selective Ca^2+^ and Mg^2+^ chelator, suggesting a pivotal role for TRP channels. Moreover the TRP channel inhibitor 2-APB is able to block TAK1 phosphorylation and, in TAK1-silenced cells, IL-1β release induced by hypotonicity is also reduced in macrophages [60].

It has been reported that membrane stretch and intracellular Ca^2+^ activate TAK1 [66,67]. After maximum stretch during cell swelling, endomembranes, derived from endosomes and lysosomes, are used to repair plasma membrane damage [68]. In macrophages, endomembrane fusion during RVD induces TRPV2 trafficking to the cell surface. TRP blockers are also effective in inhibiting monosodium urate (MSU) crystal- and nigericin-induced caspase-1 activation, but the specific involvement of TRP channels, particularly of TRPV2, in NLRP3 activation has still to be confirmed using genetic ablation in mouse models [69]. Overall, general blockers of TRP channels abolish the RVD (probably controlled by TRPM7), cell permeabilization (due to TRPV2) and caspase-1 activation.

## TLR-mediated inflammatory response is associated with TRP channel-dependent Ca^2+^ signaling and inflammasome activation in immune cells

Intracellular Ca^2+^ represents a second messenger that participates in TLR4-dependent signaling; however, how intracellular Ca^2+^ is increased in response to microbial stimuli (for example, LPS) and how it affects the inflammatory response and inflammasome generation is still only partially understood.

Several studies have indicated that TLR-mediated immune responses are associated with TRP channel-dependent Ca^2+^ signaling. Tauseef and colleagues identified a function for TRPC6 in endothelial cells (ECs). They showed that TRPC6-dependent Ca^2+^ signaling was coupled with the TLR4 signaling pathway and hence contributing to LPS-induced EC permeability and NF-κB-dependent lung inflammation [70]. Deletion of TRPC6 renders mice resistant to this endotoxin-induced barrier dysfunction and inflammation, and protects against sepsis-induced lethality. In addition, they found that LPS induces diacylglycerol in ECs, which activates TRPC6 in a TLR4-dependent manner. TRPC6-mediated Ca^2+^ entry in turn activates myosin light chain kinase, a regulator of endothelial contractility [70]. Recently, in RAW264 macrophages, it has been shown that TRPV2-mediated intracellular Ca^2+^ mobilization is involved in LPS-induced expression of inflammatory cytokines (tumor necrosis factor-α and IL-6) in a NF-κB-dependent manner [71]. Thus, it is tempting to speculate a role for TRPV2 in modulating TLR4-dependent signaling. Among TRPMs, TRPM4 is involved in LPS-induced EC death, and inhibition of TRPM4 activity protects the endothelium against LPS injury [72].

Higher levels of inflammasome components have been detected in the gingival tissues from patients with chronic periodontitis. Data obtained in differentiated THP-1 cells infected with *Porphyromonas gingivalis*, the major pathogen involved in the pathogenesis of periodontitis [73], evidenced that the microorganism induces IL-1β secretion, caspase-1 activation and pyroptotic cell death through both NLRP3 and AIM2 inflammasome activation [74]. The activation of the NLRP3 inflammasome is mediated by ATP release, P2X7 receptor and lysosomal damage. In addition, the priming signal is triggered via TLR2 and TLR4 activation precede *P. gingivalis*-induced IL-1β release [74]. Moreover, the expression of TRPV1 and TLR4 found in keratinocytes, fibroblasts, inflammatory cells and capillary endothelial cells in patients with aggressive and chronic periodontitis [75] suggests a role for TRPV1 signaling in the induction of the NLRP3 inflammasome.

Membrane TLRs respond to specific microbial components, including proteins of the virus envelopes, inducing cytokine release. The envelope protein hemagglutinins of MV (*Measles Virus*) and fusion protein of RSV (*Respiratory Syncytial Virus*) are known to interact with TLR2 and TLR4, and this effect upregulates TRP channels [76]. In particular RSV, MV and RV (*Rhinovirus*) induce TRPV1 and TRPA1 upregulation in bronchial epithelium, airway nerves and smooth muscle cells, with higher levels in asthmatic patients. Early upregulation of TRPA1 and TRPV1 expression in differentiated IMR-32 neuroblastoma cells is evidenced at 2 to 4 hours post-RV HRV-16 infection. This effect is independent of replicating virus, as virus-induced soluble factor alone is sufficient to increase TRP channel expression and augment nerve growth factor (NGF), IL-6, IL-8 and IL-1β levels in the supernatant of infected cells. By contrast, TRPM8 expression is maximal at 48 hours, and requires virus replication rather than soluble factors [76].

Further studies are required to completely understand the relationship between TLRs and TRP channels, cytokines, inflammasome activation and inflammatory response generation. The understanding of these interactions is crucial, as they may represent a novel potential therapeutic target and strategy for the treatment of chronic or acute inflammatory diseases by using TRPV1 and/or TRPA1 antagonists.

## Danger recognition pathways in the peripheral nervous system and potential role of TRP channels in the inflammasome responses

During tissue damage and pathogenic infections, release of danger signals lead to a coordinated activation of an integrated host defense formed by both peripheral neurons and immune cells which activates a complex bidirectional communication through synthesis or release of neuropeptides, neurotransmitters, hormones and cytokines. Peripheral sensory neurons are adapted to recognize danger by virtue of their sensitivity to intense mechanical, thermal and irritant chemical stimuli. In this regard, TRP ion channels are the most widely studied molecular mediators of nociception, conducting non-selective entry of cations upon activation by various noxious stimuli [77]. The anatomic position of sensory neurons at the interface with the environment, their ability to release a set of immune-acting mediators and the velocity of neural signal transduction allows the peripheral nervous system to immuno-modulate the innate and adaptive responses. By contrast, sensory neurons are very sensitive to immune mediators, which trigger and sensitize neurons [77]. Thus, neurogenic and immune-mediated types of inflammation act together as early warning devices. Nociceptor neurons in the peripheral nervous system possess many of the same molecular recognition pathways for danger as immune cells, and the line between PRRs and noxious ligand-gated ion channels is increasingly blurred.

Peripheral and terminal axons have been found to induce a rapid and local release of neural mediators during the neurogenic inflammation, independently of that produced by the immune cell system. Neuropeptides, such as calcitonin gene-related peptide (CGRP) and substance P, adrenomedullin, neurokinins A and B, vasoactive intestinal peptide, neuropeptide Y, and gastrin-releasing peptide, as well as glutamate, nitric oxide, and cytokines and chemokines [78] from nociceptors, directly attract and activate innate and adaptive immune cells [79-81]. In this regard, it has been hypothesized that substance P is able to activate the NALP1 inflammasome. Furthermore, LY303870, a NK1 receptor antagonist, blocked the upregulation of activated NALP1 inflammasome components and IL-1β and IL-18 generation in a rat fracture model of complex regional pain syndrome [82].

Another key component of the immune/sensory neuron communication system are cytokines. In early phases of inflammation, sensory neurons signal to tissue resident mast cells and dendritic cells to induce degranulation or cytokine production [79,83]. IL-1β and tumor necrosis factor-α released by innate immune cells are directly sensed during inflammation by nociceptors which express the cognate receptors [84-86]. Upon activation of cytokine receptors, signal pathways are activated in nociceptors, inducing activation of membrane proteins including TRP channels [77]. The consequent sensitization of sensory neurons means that innocuous mechanical and heat stimuli can trigger nociceptors. Interestingly, sensory neurons share many of the same pathogen and danger molecular recognition receptor pathways as innate immune cells, which enable them also to recognize pathogens [77]. As in the immune system, sensory neurons express PRRs including TLR3/4/7/9, and stimulation by their ligands induces inward currents and nociceptor sensitization [87-89]. Activation of sensory neurons by the TLR7 ligand imiquimod leads to activation of an itch-specific sensory pathway [87]. A functional expression of the IL-31RA receptor, showing a critical role in the neuroimmune link between T helper 2 cells and sensory neurons for the generation of T-cell mediated itch, was evidenced in a small subpopulation of IL-31RA(+)/TRPV1(+)/TRPA1(+) neurons [90]. In addition extracellular microRNAs (let-7b miRNA) via the TLR7 receptor that couples TRPA1 activates nociceptor neurons to elicit pain [91]. TLR4 also enhances histamine-mediated pruritus by potentiating TRPV1 activity in dorsal root ganglion (DRG) sensory neurons [92]. Furthermore, in trigeminal neurons, LPS sensitizes TRPV1 to capsaicin, which is blocked by a selective TLR4 antagonist [88]; LPS or *P. gingivalis* infection has been demonstrated to increase capsaicin-evoked release of CGRP and TRPV1 sensitization in capsaicin-sensitive nociceptors [93] and down-regulates NLRP3 inflammasome expression [94]. Respiratory virus infections can upregulate TRPV1 and TRPA1 in airway nerves with higher expression levels in asthmatic patients [76]. Overall, these data suggest the expression of a TRPV1/CGRP/TLR4 axis mediating a cross-talk between neural and immune receptors during innate immune responses through the peripheral release of neuronal signaling molecules, and the induction of infection-associated pain and itch may be partly due to direct activation of neurons by pathogen-derived factors. In addition, endotoxin by inducing TLR4 activation of TRPC6-dependent calcium signaling induces vascular permeability and inflammation in the lung [70].

Amongst the DAMP/alarmin, extracellular ATP represents the major signals recognized by sensory neurons. It is a potent danger signal that activates both peripheral neurons during cellular injury, and some evidence even suggests that neurons express components of the inflammasome molecular machinery [95]. The ATP is recognized by purinergic and TRP receptors on both nociceptor neurons [96,97]. Purinergic receptors are made up of two families: P2X receptors (ligand-gated cation channels) and P2Y receptors (G-protein coupled receptors). In nociceptor neurons, recognition of ATP through P2Y receptors contributes to nociceptor activation by TRP sensitization. Thus, in sensory neurons, the triggering of P2Y2 receptor by ATP activates the TRPV1 channel in the absence of any other stimuli [98]. In macrophages, ATP binding to P2X7 receptors induces hyperpolarization and downstream activation of the inflammasome [99].

In addition, MSU or H_2_O_2_ injection into the hind paws of rodents or application to sensory neurons increased the expression of TRPA1 and TRPV1, and enhanced cellular infiltration and IL-1β levels; these effects were blocked by TRPA1 antagonists [100]. Moreover, MSU-induced pain-like behavior, hyperalgesia, allodynia, articular edema as well as plasma extravasation, leukocyte infiltration and IL-1β production were inhibited by co-administration of a selective TRPV1 antagonist, SB366791 [101]. Mechanical hypersensitivity evoked by intraplantar injection of LPS was prevented by the TRPA1 antagonist (AP-18) and was absent in Trpa1(−/−) mice, indicating that H_2_S-mediated stimulation of TRPA1 is necessary for local pro-nociceptive effects of LPS [102].

## Inflammasome-forming NLRs in neurons of central nervous system and TRP channel involvement in neurodegenerative diseases

Inflammation in the central nervous system (CNS) has both pathogenic and protective effects depending on the clinical context. Increasing evidence suggests that inflammasomes exist in CNS cells such as neurons, astrocytes and microglia. Inflammasomes function as intracellular sensors for infectious agents as well as for host-derived danger signals and several studies have reported caspase-1 activation, IL-1β cleavage and expression of inflammasome-forming NLRs in neurons [60,103-106], as well as NLRP2 inflammasome expression in astrocytes [107]. The activation of the inflammasomes in the CNS are involved in the generation of an innate immune inflammatory response through IL-1β and IL-18 cytokine release and in cell death through the process of pyroptosis [108]. In the CNS, microglia, astrocytes and neurons express receptors for these cytokines, which thereby participate in systemic responses such as fever as well as in local inflammation within neural tissue [109,110].

Inflammasome activation is under current investigation across a broad spectrum of neurological diseases including infections, acute sterile brain injury and chronic neurodegenerative diseases. For a timely and extensive discussion on inflammasomes in CNS degenerative diseases, we direct the reader to a recent review by Walsh and colleagues, published in 2014 in *Nature Review Neuroscience* [108].

Perturbations of neuronal Ca^2+^ homeostasis are involved in the pathogenesis of several neurodegenerative disorders [111,112]. Thus, in amyotrophic lateral sclerosis/Parkinson dementia, high oxidative stress induces TRPM2 as well as TRPM7 activation leading to neurodegeneration [113]; in Parkinson's disease, dopaminergic neurons, along with coupling of mGluR1 with TRPC1/3/5 channels, results in alteration of neuronal activity [114,115]. TRPV1 and TRPV2 agonists diminished dopamine depletion and tyrosine hydroxylase deficits caused by the neurotoxin 6-hydroxydopamine in the striatum [116] and overexpression of TRPC1 increased dopaminergic neuron survival following MPP neurotoxin administration [117]. TRPM7-associated Mg^2+^-inhibited cation channel activity plays a role in neurodegenerative variations associated with Alzheimer's disease (AD) [118]; in AD, presenilin-2 negatively influences the TRPC6-dependent calcium entry [119] and inhibition of TRPM2 by siRNA reduces amyloid-β toxicity, suggesting a role for TRPM2 in neuronal cell death [120]. By contrast, in AD activation of TRPM7 leads to zinc accumulation and zinc-induced neurotoxicity [121]. TRPM2 and TRPM7 contribute to ischemic neural death and dysfunction, and their inhibition or gene silencing increased neuronal survival during ischemia [122].

Inflammasome proteins and even the functional machinery of inflammasomes have been linked to cell death in several cell types [123]. Tissue damage caused by oxidative stress has been found to play a pathogenetic role in several chronic neurodegenerative diseases such as AD and Parkinson’s disease. Substantial evidence supports the conclusion that TRP channels, in particular the TRPM2 channel, link cell death induced by oxidative stress [120] with the NLRP3 inflammasome activation [124].

Amyloid-β, the chief constituent of senile plaques in Alzheimer’s disease, was the first molecule associated with a neurodegenerative disease to be shown to activate the inflammasome (for example, NLRP3) [125,126]. Amyloid β-peptide and H_2_O_2_ have been found to induce cell death in primary cultures of rat striatal cells which express TRPM2 [120]. Four physiological splice variants of TRPM2 have been identified at present: TRPM2-L (full length or wild-type), TRPM2-S, TRPM2-ΔN and TRPM2-ΔC [127]. In striatal cells, the dominant negative splice variant TRPM2-S inhibits both amyloid β-peptide and H_2_O_2_-induced increase in [Ca^2+^]_i_ and protects cell viability. Moreover, reduction in endogenous TRPM2 levels by RNA interference significantly enhances cell viability after β-peptide and H_2_O_2_ treatment.

Overall, these data provide significant support to the conclusion that TRPM2 is involved in oxidative stress-induced injury to striatal cells and, through activation by amyloid β-peptide, may be involved in the pathogenesis of AD [127].

Further studies may be required to completely assess the involvement of TRP channel signaling in inflammasome activation in neurodegenerative diseases.

## Conclusions

Overall, the findings recently reported strongly suggest an important role played by ion channels belonging to the TRP channel families that mediates interchanged and bidirectional contact and intracellular signals with known PAMPs and DAMPs in inflammasome activation in immune and sensory nervous cells (Table 1). The development of new drugs to target inflammasome functions are now promising possibilities for future therapeutic interventions in neurological diseases. These results highlight the therapeutic potential of targeting different TRP channels by using TRP antagonists, or different molecules able to inhibit the TRP-induced signaling pathway, in autoinflammatory and metabolic disorders associated with undesirable activation and generation of the inflammasome.

**Table 1:** **DAMP/PAMP recognition by TRP channels and PRRs triggers the inflammasome activation in immune and neuron cells**

**DAMP/PAMP**	**TRP channel/s**	**Immune cells**	**Non-immune cells**	**Type of inflammasome**	**Reference**
Acid pH	TRPV1	Macrophages		NLRP3	[39]
Cold	TRPM8	PBMCs	Neurons	?	[44]
Liposome/crystals	TRPM2	Phagocytes		NLRP3	[123]
RVD	TRPV2	Macrophages		NLRP3	[128]
	TRPM7	Macrophages		NLRP3	[60]
Osmotic stress	TRPV2	Dendritic cells		NLRP3	[16]
MSU/alum	TRPV2	Monocytes		NLRP3	[59]
	TRPA1		Sensory neurons	?	[100]
	TRPV1		Neurons	?	[100]
ATP	P2Y2/TRPV1		Sensory Neurons	?	[98]
miRNA	TLR7/TRPA1		Sensory neurons	?	[91]
Mechanical stretch	TRPC3	Monocytes		?	[129]
Ca^2+^	TLR4-TRPC6		Endothelial cells	?	[70]
RV	TRPA1		Neuroblastomas	NLRP3	[76]
	TRPV1				
	TRPM8				
RSV/MS	TLR2/4-TRPV1		Epithelial cells	NLRP3	[76]
	TLR2/TRPA1		Smooth muscle cells	NLRP3	[76]
	TLR2/4-TRPM8		Neuroblastomas	NLRP3	[76]
PG	TLR2/4-TRPV1	Monocytes		NLRP3	[74]
		Macrophages		AIM2	[74]
	TLR4-TRPV1		Sensory neurons	?	[88,93]
LPS	TLR4/TRPC6		Sensory Neurons	?	[70]

?, Suggested, but not yet identified; AIM2, absent in melanoma 2; DAMP, danger-associated molecular pattern; LPS, lipopolysaccharide; MSU, monosodium urate; MV, *Measles virus*; NLRP3, NOD-like receptor family, pyrin domain-containing 3; P2Y2, Purinergic receptor P2Y G-protein coupled 2; PAMP, pathogen-associated molecular pattern; PBMC, peripheral blood mononuclear cell; PG, *Porphyromonas gingivalis*; RV, *Rhinovirus*; RSV, *Respiratory syncytial virus*; RVD, regulatory volume decrease; TLR, Toll-like receptor; TRP, transient receptor potential; TRPM2, TRP Melastatin type-2; TRPM7, TRP Melastatin type 7; TRPM8, TRP Melastatin type-8; TRPA1, TRP Ankyrin-like protein with transmembrane-like domain 1; TRPC3, TRP Canonical type-3, TRPV1, TRP vanilloid type-1; TRPV2, TRP Vanilloid type-2.

## Abbreviations

AD: Alzheimer's disease
CGRP: calcitonin gene-related peptide
CNS: central nervous system
DAMP: danger-associated molecular pattern
EC: endothelial cell
ER: endoplasmic reticulum
IL: interleukin
LPS: lipopolysaccharide
MSU: monosodium urate
NF: nuclear factor
NLR: NOD-like receptor (nucleotide-binding oligomerization domain receptor)
PAMP: pathogen-associated molecular pattern
PRR: pattern recognition receptor
ROS: reactive oxygen species
RVD: regulatory volume decrease
TLR: Toll-like receptor
TRP: transient receptor potential
